# Interaction of nutritional status and diabetes on active and latent tuberculosis: a cross-sectional analysis

**DOI:** 10.1186/s12879-019-4244-4

**Published:** 2019-07-16

**Authors:** Rachel W. Kubiak, Sonali Sarkar, C. Robert Horsburgh, Gautam Roy, Mario Kratz, Ayiraveetil Reshma, Selby Knudsen, Padmini Salgame, Jerrold J. Ellner, Paul K. Drain, Natasha S. Hochberg

**Affiliations:** 1 Department of Epidemiology, University of Washington, 1959 NE Pacific Street, Health Sciences Building, Box 357236, Seattle, WA 98195 USA; 2 Department of Preventive and Social Medicine, Jawaharlal Institute of Postgraduate Medical Education and Research, Pondicherry, India; 3 Department of Epidemiology, Boston University School of Public Health, Boston, USA; 4 Section of Infectious Diseases, Department of Medicine, Boston University School of Medicine, Boston, USA; 5 Division of Metabolism, Endocrinology, and Nutrition, Department of Medicine, University of Washington, Seattle, USA; 6 Cancer Prevention Program, Division of Public Health Sciences, Fred Hutchinson Cancer Research Center, Seattle, USA; 7 Center for Emerging Pathogens, Department of Medicine, Rutgers-New Jersey Medical School, Newark, USA; 8 Boston Medical Center, Boston, USA; 9 Department of Global Health, University of Washington, Seattle, USA; 10 Department of Medicine, University of Washington, Seattle, USA

**Keywords:** Body mass index, Cross-sectional analysis, Diabetes mellitus, Latent tuberculosis, Prevalence, Tuberculosis, Underweight, India

## Abstract

**Background:**

Malnutrition and diabetes are risk factors for active tuberculosis (TB), possible risk factors for latent TB infection (LTBI), and may interact to alter their effect on these outcomes. Studies to date have not investigated this interaction.

**Methods:**

We enrolled 919 newly diagnosed active TB patients and 1113 household contacts at Primary Health Centres in Puducherry and Tamil Nadu, India from 2014 to 2018. In cross-sectional analyses, we used generalized estimating equations to measure additive and multiplicative interaction of body mass index (BMI) and diabetes on two outcomes, active TB and LTBI.

**Results:**

Among overweight or obese adults, active TB prevalence was 12-times higher in diabetic compared to non-diabetic participants, 2.5-times higher among normal weight adults, and no different among underweight adults (*P* for interaction < 0.0001). Diabetes was associated with 50 additional active TB cases per 100 overweight or obese participants, 56 per 100 normal weight participants, and 17 per 100 underweight participants (*P* for interaction < 0.0001). Across BMI categories, screening 2.3–3.8 active TB patients yielded one hyperglycemic patient. LTBI prevalence did not differ by diabetes and BMI*diabetes interaction was not significant.

**Conclusions:**

BMI and diabetes are associated with newly diagnosed active TB, but not LTBI. Diabetes conferred the greatest risk of active TB in overweight and obese adults whereas the burden of active TB associated with diabetes was similar for normal and overweight or obese adults. Hyperglycemia was common among all active TB patients. These findings highlight the importance of bi-directional diabetes-active TB screening in India.

**Electronic supplementary material:**

The online version of this article (10.1186/s12879-019-4244-4) contains supplementary material, which is available to authorized users.

## Background

Active tuberculosis (TB) disease is a major cause of morbidity and the leading infectious cause of mortality globally [1]. Achieving the World Health Organization (WHO) End TB Strategy goal of TB incidence < 10/100,000 population by 2035 will require a multi-pronged approach including providing adequate clinical care for comorbidities that are risk factors for active TB disease, and targeted screening and treatment of latent TB infection (LTBI) for those at high risk of progressing to active disease [2, 3]. Diabetes and malnutrition, known risk factors for active TB, have been recognized as two important factors that could prevent achieving the global target of reducing TB incidence by 2035 [4]. Diabetes increases the risk of active TB by approximately three-fold [5, 6]. Conversely, for each unit increase in body mass index (BMI), the risk of TB decreases by 13.8% on average [7]. Whereas obesity has a direct protective effect on active TB, it is also a risk factor for diabetes, which may negate protection conferred from high BMI [8]. The rising prevalence of diabetes may be contributing to persistently high TB incidence in high TB burden countries, outweighing the protective effect of high BMI [9, 10]. However, the interaction between BMI and diabetes has not been previously estimated. Improved understanding of the populations most at risk for active TB at national and sub-national levels is essential to implementing the WHO End TB Strategy effectively [2, 3].

India has high dual burdens of TB and diabetes. Approximately 74 million Indian adults have diabetes (10.4%) and this number is expected to nearly double by 2045 [11]. Among 2.7 million incident TB cases in India, 15% may be attributable to diabetes and 32–62% may be attributable to malnutrition [1, 12, 13]. These diseases are associated with personal catastrophic health expenditures in vulnerable populations, which may be partially mitigated through proactive screening practices and preventative care [14]. Bi-directional diabetes-TB screening is recommended by the WHO and in Indian national guidelines [15, 16]. In India, all TB patients should be screened for random blood glucose (RBG) ≥140 mg/dL [15]. At each diabetes clinic visit, patients should be screened for TB symptoms and referred for TB testing if positive [15]. Given the challenge of providing quality care to this large and growing diabetic patient population, identifying those most likely to have active TB could help to focus limited resources.

The relationship between nutritional status, diabetes, and LTBI is less clear. LTBI is chronic *Mycobacterium tuberculosis* (MTB) infection without evidence of clinical disease affecting one quarter of the world’s population, of whom 5–15% will develop active TB over their lifetime [1]. A meta-analysis found no difference in the odds of LTBI among underweight compared to normal weight adults [17]. Diabetes is associated with a higher odds of LTBI by up to 2-fold but studies have had mixed results [18–20].

We tested for additive and multiplicative interaction between BMI and diabetes on two separate outcomes, active TB and LTBI, in an observational study of TB patients and their household contacts in south India. Here, we present both the individual and combined effects of BMI and diabetes on active TB disease and LTBI in order to estimate the relative prevalence and burden of these outcomes attributable to diabetes overall and within BMI strata. We also estimated the number of TB patients needed to screen by BMI category in order to identify one instance of hyperglycemia.

## Methods

### Study population

We conducted cross-sectional analyses of newly diagnosed active TB patients and their household contacts in southern India as part of the Regional Prospective Observational Research for Tuberculosis (RePORT)-India Consortium [12]. Enrolment began in Pondicherry in May 2014, and in two districts of Tamil Nadu, Cuddalore and Vilupuram, in August 2014 and November 2015, respectively.

Acid-fast bacilli sputum smear positive TB patients were recruited at Revised National TB Control Program District Microscopy Centres and Primary Healthcare Centres. Eligible TB patients for this RePORT-India site were ≥ 6 years of age; able to provide sputum for a confirmatory culture; enrolled in directly observed therapy, short-course at their local clinic; and willing to be tested for HIV. TB patients with ≥3 doses of anti-TB therapy at enrolment, a history of TB disease or treatment, or a multi-drug resistant TB contact were excluded.

Household contacts were eligible for enrolment if they had lived with the TB patient for at least the previous 3 months, were ≥ 6 years old, had no prior TB diagnosis, no known contact with a multi-drug resistant TB patient, and were willing to be tested for LTBI.

### Ethics, consent and permissions

All participants enrolled in the study were willing and able to provide written informed consent or assent in conjunction with parental/guardian consent if < 18 years. The study protocol was approved by the Jawaharlal Institute of Postgraduate Medical Education and Research Ethics Committee and Scientific Advisory Committee, and the Institutional Review Boards at Boston University Medical Campus and Rutgers-New Jersey Medical School.

### Study procedures

At each participant’s enrolment visit, research teams collected demographic and health information and measured participants’ height and weight to calculate a BMI. Active TB patients provided a sputum sample at enrolment for Löwenstein-Jensen and liquid mycobacterial growth indicator tube cultures (Becton Dickinson, USA) and received an RBG test by finger stick. The clinics performed HIV testing as part of the standard of care.

At household contact enrolment, which occurred primarily at the household, the study nurse injected 0.5 ml of purified protein derivative into the intradermal layer as a tuberculin skin test (TST) (Span Diagnostics/Arkray Healthcare, India). To determine LTBI status, the study nurse measured induration within 5 days and the majority within 3 days. Household contacts with TB symptoms and a positive skin test were asked to provide a sputum sample for AFB smear and culture. Screening for diabetes among household contacts using RBG began in April 2016. Individuals were questioned regarding a history of renal failure. All household contacts were followed for 1 year; symptom screens (and sputum testing if indicated) were performed to identify incident active TB disease.

### Statistical analyses

We used standard BMI categories for the Indian Asian population of underweight (< 18.5 kg/m^2^), normal weight (18.5–22.9 kg/m^2^), and overweight or obese (≥23.0 kg/m^2^) [21]. A patient was considered to have confirmed diabetes if they reported a prior clinical diagnosis of diabetes. Active TB was defined as sputum culture-positive for *MTB* either by solid or liquid culture. Household contact LTBI was defined as TST induration ≥5 mm [22].

We excluded participants enrolled as household contacts who tested culture-positive for MTB following a positive symptom screen, participants enrolled as cases who were not MTB culture-positive, participants < 18 years of age, and participants missing both self-reported diabetes status and an RBG measurement.

Among active TB patients, we estimated the number needed to screen (NNS) for elevated RBG as one over the prevalence of RBG ≥140 mg/dL for each BMI category, as per national guidelines for diabetes screening [15]. We estimated the NNS both for those who did not report a prior diagnosis of diabetes and for those who did. To estimate the overall relationship between diabetes and active TB disease, we calculated unadjusted and adjusted prevalence ratios using generalized estimating equations (GEE) with a log link and binomial distribution, or if the model failed to converge, a Poisson distribution with robust standard errors [23, 24]. We accounted for clustering at the family-level in all models using an exchangeable correlation matrix [23]. For the adjusted model, we decided a priori to control for age (years), sex, BMI category, smoking (current/not current), and hazardous alcohol use as per the Alcohol Use Disorders Identification Test (AUDIT)-C questionnaire (≥3 for women and ≥ 4 for men) [25].

To evaluate multiplicative and additive interaction of diabetes and BMI on active TB disease, we calculated BMI-diabetes stratum-specific prevalence ratios and prevalence differences, respectively, adjusting for age and sex [26]. We chose normal BMI, non-diabetic household contacts who by definition did not have active TB disease as the reference group. We used GEE with a binomial distribution or Poisson distribution with robust standard errors if needed, and a log-link to estimate the prevalence ratios or identity-link to estimate prevalence differences [23, 24]. We employed the same GEE approach to estimate the overall relative unadjusted and adjusted risks of LTBI among household contacts with diabetes compared to those without diabetes. We used non-diabetic household contacts with a normal BMI and no LTBI as the reference group.

We performed two sensitivity analyses of the overall relationship of diabetes with active and latent TB: 1) defining diabetes as RBG ≥200 mg/dL or self-report of a prior clinical diagnosis of diabetes, and 2) excluding participants with a moderately abnormal RBG of 140–199 mg/dL [15]. We used SAS version 9.4 (Cary, NC).

## Results

Of the 2032 participants included in analyses, 919 were active TB patients and 1113 were household contacts without active TB disease. Compared to household contacts, TB patients were more often male (79% versus 35%), older (mean age 45 versus 37 years), and more commonly engaged in hazardous alcohol use (46% versus 6%) (Table 1). The majority (61%) of TB patients were underweight compared to only 16% of household contacts.

**Table 1:** Baseline characteristics of adult tuberculosis cases and their household contacts in southern India (*n* = 2032)

	Active TB Case	Household Contact
	(*n* = 919)	(*n* = 1113)
	n (%) or mean ± std	n (%) or mean ± std
Sociodemographics		
Male (*n* = 1969)	729 (79.3)	392 (35.2)
Age (years)	44.9 ± 14.0	36.8 ± 14.4
Years of schooling	6.9 ± 4.7	8.0 ± 5.1
Married/Living together	673 (73.2)	702 (63.1)
Household income ≤5,000 rupees (*n* = 1817)^a^	465 (50.6)	395 (44.0)
Hazardous alcohol use	426 (46.4)	70 (6.3)
Current smoker	215 (23.4)	107 (9.6)
Body Mass Index (kg/m^2^)		
Underweight (< 18.5)	564 (61.4)	182 (16.4)
Normal (18.5–22.9)	266 (28.9)	387 (34.8)
Overweight/obese (≥23.0)	89 (9.7)	461 (48.9)
Diabetes measurements		
Prior diabetes diagnosis	296 (32.2)	69 (6.2)
Random blood glucose (mg/dL) (n = 1635)	180.8 ± 103.4	132.0 ± 63.8
Random blood glucose ≥200 mg/dL (*n* = 1635)	263 (28.6)	58 (5.2)
Prior diabetes diagnosis or random blood sugar ≥200 mg/dL	343 (37.3)	94 (8.5)
Tuberculosis testing		
Tuberculin skin test positive	NA	605 (54.4)
Tuberculin skin test induration (mm)^b^	NA	7 (2–11)
Days to MGIT positivity (*n* = 916)	8.5 ± 4.1	NA
Other Comorbidities		
Self-reported history of renal failure	1 (0.1)	3 (0.3)
HIV positive	3 (0.3)	NA

*MGIT* mycobacterial growth indicator tubes, *NA* Not Available, *Std* standard deviation

^a^ Equivalent to ~ 75 USD

^b^ Median (interquartile range)

Overall, 365 (18%) participants reported a prior diabetes diagnosis, and diabetes was more common among active TB patients than their household contacts (32% versus 6%). Of the 344 diabetic participants who also received RBG testing, 72% had an RBG ≥200 mg/dL whereas 66/1155 (6%) of those who had not been diagnosed with diabetes had RBG ≥200 mg/dL. The majority (79%) of participants with known diabetes reported using oral medication to control their diabetes in the past month and 35% were overweight or obese. Of those with RBG ≥200 mg/dL but no prior diabetes diagnosis, 32% were overweight or obese. The proportion of underweight, normal weight, and overweight or obese participants with diabetes was 11% (82/746), 25% (157/653), and 20% (126/633) respectively.

Among TB patients overall, 49% had an RBG ≥140 mg/dL and the NNS ranged from 1.2 to 2.9 (Table 2). Of those who reported a prior diabetes diagnosis, 36% were diagnosed in the year prior to their TB diagnosis, 91% had elevated RBG, and NNS did not vary by BMI category.

**Table 2:** Number of active tuberculosis patients screened to identify one instance of elevated random blood glucose (*n* = 915)

		Body mass index (kg/m^2^)		
		< 18.5	18.5–22.9	≥23.0
Overall	Total (n)	562	264	89
	RGB ≥140 (%)	34.9	67.8	82
	Number needed to screen	2.9	1.5	1.2
No prior diabetes	Total (n)	484	121	18
	RGB ≥140 (%)	26.2	39.7	44.4
	Number needed to screen	3.8	2.5	2.3
Diabetes diagnosis	Total (n)	78	143	71
	RGB ≥140 (%)	88.5	91.6	91.6
	Number needed to screen	1.1	1.1	1.1

*RGB* random blood glucose (mg/dL)

The adjusted prevalence of active TB was 2.13-times higher among adults with diabetes compared to those without diabetes (95% confidence interval [CI] 1.95, 2.33) (Table 3). Being underweight was associated with a higher prevalence of TB (adjusted prevalence ratio [aPR] 1.59; 95% CI 1.45, 1.75) whereas being overweight or obese was associated with lower TB prevalence (aPR 0.40; 95% CI 0.32, 0.49). Comparing those with diabetes to those without diabetes, the prevalence of active TB was 1.04-times higher among underweight participants, 2.45-times higher among normal weight participants, and 12-times higher among overweight or obese participants (*P* for interaction < 0.0001) (Table 4). Among overweight or obese participants without diabetes, the adjusted risk of active TB was 0.17-times (95% CI 0.11, 0.27) that of normal weight adults without diabetes, but this protective association was not observed among overweight or obese participants with diabetes (aPR 1.73, 95% CI 1.38, 2.17).

**Table 3:** Prevalence of latent tuberculosis infection and active tuberculosis disease among diabetic compared to non-diabetic adults^a^

	LTBI (*n* = 1113)			TB (*n* = 2032)		
	n (%)	PR (95% CI)	aPR (95% CI)^b^	n (%)	PR (95% CI)	aPR (95% CI)^b^
No history of diabetes	561/1044 (53.7)	1.00 (Referent)	1.00 (Referent)	623/1667 (37.4)	1.00 (Referent)	1.00 (Referent)
Prior diabetes diagnosis	44/69 (63.8)	1.24 (1.04, 1.48)	1.20 (0.99, 1.45)	296/365 (81.1)	3.83 (2.01, 7.30)	2.13 (1.95, 2.33)
Sensitivity analyses						
RBG < 200	553/1019 (54.2)	1.00 (Referent)	1.00 (Referent)	576/1595 (36.1)	1.00 (Referent)	1.00 (Referent)
RBG ≥ 200 or prior diabetes diagnosis	52/94 (55.3)	1.04 (0.86, 1.25)	1.00 (0.82, 1.21)	343/437 (78.5)	2.17 (1.90, 2.48)	2.06 (1.90, 2.23)
RBG < 140	517/928 (55.7)	1.00 (Referent)	1.00 (Referent)	440/1368 (32.2)	1.00 (Referent)	1.00 (Referent)
RBG ≥ 200 or prior diabetes diagnosis	52/94 (55.3)	1.01 (0.84, 1.22)	0.97 (0.80, 1.18)	343/437 (78.5)	2.47 (2.25, 2.71)	2.19 (2.00, 2.39)

*aPR* adjusted prevalence ratio, *CI* confidence interval, *LTBI* latent tuberculosis infection, *PR* prevalence ratio, *RBG* random blood glucose (mg/dL), *TB* tuberculosis

^a^ All models account for clustering at the family-level with an exchangeable correlation matrix. For LTBI, the referent group is LTBI-negative household contacts. For TB, the reference group is all household contacts

^b^ Adjusted for age, sex, body mass index category, smoking, and hazardous alcohol use

**Table 4:** Relative and additive effect modification of diabetes on active tuberculosis prevalence by body mass index^a^

	BMI Category (kg/m^2^)											
	< 18.5				18.5–22.9				≥23.0			
	TB cases/total	%	aPR (95% CI)	aPD (95% CI)	TB cases/ total	%	aPR (95% CI)	aPD (95% CI)	TB cases/ total	%	aPR (95% CI)	aPD (95% CI)
No history of diabetes	484/664	72.9	2.44 (2.09, 2.85)	0.40 (0.34, 0.45)	121/496	24.4	1.00 (Referent)	0.00 (Referent)	18/507	3.6	0.17 (0.11, 0.27)	−0.16 (−0.20, −0.13)
Prior diabetes diagnosis	80/82	97.6	2.38 (1.98, 2.86)	0.61 (0.53, 0.68)	145/157	92.4	2.45 (2.05, 2.92)	0.56 (0.49, 0.63)	71/126	56.4	1.73 (1.38, 2.17)	0.24 (0.15, 0.34)
Effect of diabetes within BMI strata			1.04 (0.93, 1.16)	0.17 (0.12, 0.23)			2.45 (2.05, 2.92)	0.56 (0.49, 0.63)			12.01 (6.98, 20.71)	0.50 (0.41, 0.59)

*aPD* adjusted prevalence difference, *aPR* adjusted prevalence ratio, *BMI* body mass index, *CI* confidence interval, *TB* tuberculosis

*P* for interaction additive and relative scales < 0.0001

^a^ Adjusted for age and sex, and accounting for clustering at the family-level with an exchangeable correlation matrix

**Fig. 1:**
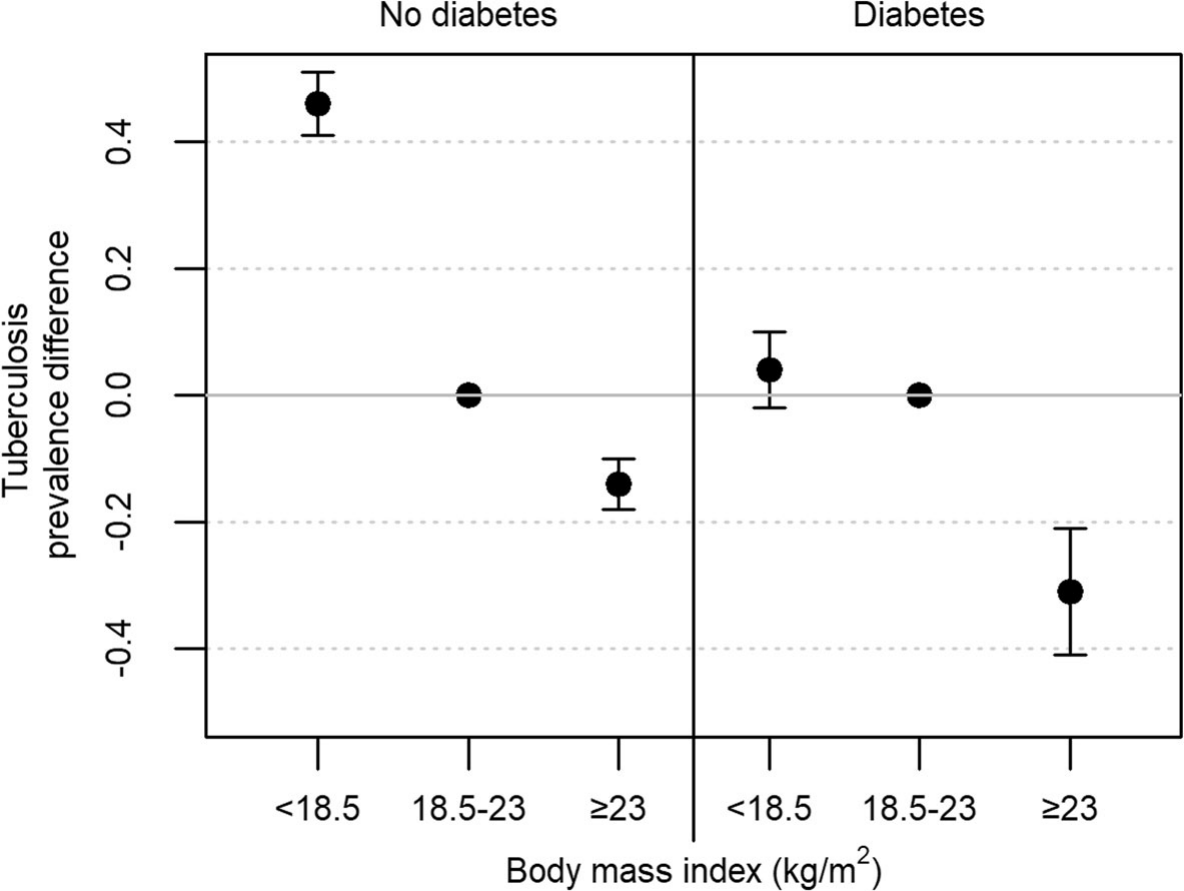
Adjusted difference in prevalence of active tuberculosis between body mass index categories, by diabetes status. Legend: Points represent the adjusted prevalence difference and vertical bars represent 95% confidence intervals. Adjusted for age and sex, and accounting for clustering at the family-level with an exchangeable correlation matrix

On the additive scale, diabetes was associated with an estimated 17 additional TB patients per 100 underweight participants, 56 patients per 100 normal weight participants, and 50 patients per 100 overweight or obese participants (*P* for interaction < 0.0001) (Table 4). Among those without diabetes, the prevalence of active TB was higher among underweight participants and lower among overweight or obese participants compared to those of normal weight (Fig. 1).

The prevalence of LTBI was no different among household contacts with diabetes (aPR 1.20; 95% CI 0.99, 1.45) or in sensitivity analyses (Table 2). Diabetes was associated with LTBI among underweight participants (aPR 1.97; 95% CI 1.32, 2.93; aPD 0.49; 95% CI 0.27, 0.70) (Additional file 1: Table S1 and S2). Interaction with BMI was not significant on the relative or additive scale (*P* for both interactions > 0.1).

## Discussion

We present evidence of both additive and multiplicative interaction between BMI and diabetes in active TB at the time of diagnosis in this southern Indian cohort. Our findings provide support for current Indian national guidelines recommending bi-directional screening of all diabetes patients for active TB and all TB patients for diabetes irrespective of BMI. Among active TB patients, the NNS to yield one instance of hyperglycemia was low for all BMI categories. The highest relative risk of active TB from diabetes was among overweight and obese adults, whereas the greatest burden of active TB disease due to diabetes was among adults of normal weight. Low BMI was also associated with active TB. LTBI was not associated with prior diabetes diagnosis alone and we found no evidence of BMI-diabetes interaction.

The prevalence of diabetes among TB patients in our study population was similar to previously reported estimates in India [27, 28]. Nearly all TB patients with a prior diabetes diagnosis had elevated RBG suggesting an important opportunity to provide diabetes counselling, referral to diabetes care, and glucose monitoring. Of those who did not report a diabetes diagnosis, 29% still had elevated RBG. Incident TB is associated with hyperglycemia that may resolve over the course of TB treatment independent of diabetes interventions, but may also be a prognostic marker of poor short-term outcomes and longer-term elevated risk of diabetes [6, 29–32].

Our findings consistently support the inverse association of BMI with active TB risk and increased risk of TB from diabetes [5, 7]. Our observation of highly significant multiplicative and additive BMI-diabetes interaction on active TB adds to the body of literature by identifying for whom and to what extent diabetes and BMI are associated with newly diagnosed active TB in this resource-limited setting. Multiplicative interaction is less heterogeneous than additive interaction, which is more closely tied to the size of the burden of disease in the study population [33, 34]. The additive scale, estimating the difference-in-differences, is less commonly reported but is appropriate for estimating public health impacts and identifying high-risk groups to inform resource allocation [26, 33]. We maximize the programmatic relevance by identifying the high burden of diabetes-associated TB among both normal weight and overweight or obese patients, and the high proportion of TB patients with hyperglycemia at all levels of BMI. Bi-directional screening could lead to earlier diagnosis of both conditions and improved disease management, but additional research is needed to optimize integrated care and identify the most cost-effective screening methods.

Prior studies suggest diabetes may modestly increase the risk of LTBI but the evidence is mixed [18, 19, 35]. A recent meta-analysis found the odds of LTBI was higher among diabetic patients although the effect size was small (1.18, 95% CI 1.06, 1.30) [18]. However, LTBI was not associated with diabetes in a prospective cohort study or recent cross-sectional analyses of another Indian cohort [18, 20]. Similarly, in both unadjusted and adjusted models, we found no statistically or clinically significant association of LTBI with prior diabetes diagnosis, or in sensitivity analyses with elevated RBG. These findings add to the evidence that diabetes is not a significant risk factor for LTBI in India and instead suggest diabetes increases the risk of TB activation.

Mechanistic studies provide biological plausibility for increased risk of both MTB acquisition and progression to TB disease in the setting of diabetes and malnutrition. Murine models demonstrate defective innate and adaptive immune responses in the presence of diabetes [35, 36]. In diabetic mice challenged with aerosolized MTB, the priming of the adaptive immune response is delayed resulting in impaired local immune response in the lung and likely increased susceptibility to TB disease [36]. Human studies have found alterations in central memory T cells, effector memory T cells, and T regulatory cells among TB patients with diabetes [37]. Similarly, malnutrition affects a range of immune responses from macrophage phagocytosis and activation to T cell response and IFNγ production that results in increased TB risk [38]. A systematic review of cohort studies identified an inverse log-linear relationship between BMI and active TB [7], but additional studies of the biological mechanisms involved in the interaction between BMI and diabetes are needed.

Our study has several strengths in addition to estimating interaction on both the multiplicative and additive scale. We present prevalence ratios, which are more intuitive, conservative, and consistent than odds ratios, which do not approximate a risk ratio when the outcome is common [24, 39]. We also had thorough ascertainment of active TB using sputum culture for both household contacts and TB patients. Household contacts with a positive TST and TB symptoms were tested for active TB at enrolment and all household contacts were followed for 1 year, a high-risk period, to identify incident active TB.

Our study also has several limitations. First, our data were cross-sectional, precluding causal inference. Some active TB patients recently diagnosed with diabetes may in fact have transient hyperglycemia caused by TB, biasing results away from the null. Malnutrition is an established risk factor for active TB, but unexpected weight loss is also a common effect [7]. For these analyses estimating the burden of hyperglycemia and diabetes for different BMI categories at the time of TB diagnosis, the order of events is less critical. Second, our results are most relevant to adults because we only included participants ≥18 years in our analyses and type 2 diabetes, which accounts for more than 90% of cases, tends to develop in adulthood [11]. Third, only two underweight household contacts had diabetes and both had LTBI, so the reliability of our estimates in this low BMI category is limited. Fourth, bacille Calmette-Guérin vaccination reduces the specificity of TST. However, this effect wanes over 10 years and the vaccination is given in infancy so we expect negligible impact in this adult population [22, 40]. Finally, we compared active TB patients to household contacts, not community-based controls. We believe household contacts are more representative of the lower socio-economic population from which the cases were drawn. Additionally, we took a conservative statistical approach using prevalence estimates and accounting for family-level similarities with an exchangeable correlation matrix.

Our findings may not be generalizable beyond Indian Asian populations who have a higher likelihood of developing diabetes at every BMI level [41]. We observed a high proportion of diabetes among those of normal or even low BMI, in line with the “*thin-fat phenotype*” observed in India wherein Indians have more body fat and central obesity for each BMI category compared to Caucasians and black Africans [41].

## Conclusions

Our analyses of multiplicative and additive interaction between diabetes and BMI on active TB highlight the high prevalence of diabetes among active TB patients at all levels of BMI in this south Indian cohort. Malnutrition as measured by BMI was also associated with a higher burden of TB, primarily among those without diabetes. BMI and diabetes were not major risk factors for LTBI. Additional research is needed to better understand the biological mechanisms involved, optimize timing of diabetes testing and clinical care for diabetic or hyperglycemic TB patients, assess the cost-effectiveness of bi-directional screening, and estimate the public health impact of diabetes and obesity on active TB in other regions.

## Additional file

Additional file 1: **Table S1.** and **Table S2.** for adjusted prevalence ratios and difference of latent TB infection by BMI and diabetes status. (DOCX 18 kb)

## Data Availability

The datasets analyzed are available from the corresponding author on reasonable request.

## Abbreviations

aPD: Adjusted prevalence difference
aPR: Adjusted prevalence ratio
BMI: Body mass index
GEE: Generalized estimating equations
LTBI: Latent tuberculosis infection
MTB: *Mycobacterium tuberculosis*
NNS: Number needed to screen
RBG: Random blood glucose
RePORT: Regional Prospective Observational Research for Tuberculosis
TB: Tuberculosis
TST: Tuberculin skin test
