# Supplementary material for: Interaction of nutritional status and diabetes on active and latent tuberculosis: a cross-sectional analysis
Source: BMC Infect Dis. 2019 Jul 16;19:627. doi: 10.1186/s12879-019-4244-4 (PMC6636094; doi:10.1186/s12879-019-4244-4)
Supplement: Supplementary file 1 — Table S1. and Table S2. for adjusted prevalence ratios and difference of latent TB infection by BMI and diabetes status. (DOCX 18 kb) [file 12879_2019_4244_MOESM1_ESM.docx]

**Interaction of nutritional status and diabetes on active and latent tuberculosis**

Rachel W. Kubiak, Sonali Sarkar, C. Robert Horsburgh, Jr., Gautam Roy, Mario Kratz, Ayiraveetil Reshma, Selby Knudsen, Padmini Salgame, Jerrold J. Ellner, Paul K. Drain and Natasha S. Hochberg.

Additional file 1: Tables S1 and S2

**Table S1. Relative scale for effect modification of diabetes on latent tuberculosis infection prevalence by body mass index category in south India^a^**

|  | **BMI Category** | | | | | | | | | | | | | |
| --- | --- | --- | --- | --- | --- | --- | --- | --- | --- | --- | --- | --- | --- | --- |
|  | **<18.5** | | | |  | **18.5-22.9** | | | |  | **≥23** | | | |
|  | **LTBI/Total** | **%** | **aPR** | **95% CI** |  | **LTBI/Total** | **%** | **aPR** | **95% CI** |  | **LTBI/Total** | **%** | **aPR** | **95% CI** |
| No history of diabetes | 103/180 | 57.2 | 1.18 | 0.99, 1.40 |  | 180/375 | 48.0 | **1.00** | **Referent** |  | 278/489 | 56.9 | 1.12 | 0.99, 1.27 |
| Prior diabetes diagnosis | 2/2 | 100 | 1.55 | 1.13, 2.12 |  | 7/12 | 58.3 | 1.04 | 0.62, 1.76 |  | 35/55 | 63.6 | 1.24 | 0.97, 1.58 |
| Effect of diabetes within strata of BMI |  |  | 1.97 | 1.32, 2.93 |  |  |  | 1.04 | 0.62, 1.76 |  |  |  | 1.14 | 0.90, 1.43 |

aPR, adjusted prevalence ratio; BMI, body mass index; CI, confidence interval; LTBI, latent tuberculosis infection.

*P* for interaction 0.109

^a^ Adjusted for age and sex, and accounting for clustering at the family-level with an exchangeable correlation matrix.

**Table S2. Additive scale for effect modification of diabetes on latent tuberculosis infection prevalence by body mass index category in south India^a^**

|  | **BMI Category** | | | | | | | | | | | | | |
| --- | --- | --- | --- | --- | --- | --- | --- | --- | --- | --- | --- | --- | --- | --- |
|  | **<18.5** | | | |  | **18.5-22.9** | | | |  | **≥23** | | | |
|  | **LTBI/Total** | **%** | **aPD** | **95% CI** |  | **LTBI/Total** | **%** | **aPD** | **95% CI** |  | **LTBI/Total** | **%** | **aPD** | **95% CI** |
| No history of diabetes | 103/180 | 57.2 | 0.09 | 0.00, 0.19 |  | 180/375 | 48.0 | **0.00** | **Referent** |  | 278/489 | 56.9 | 0.05 | -0.01, 0.11 |
| Prior diabetes diagnosis | 2/2 | 100 | 0.36 | 0.19, 0.53 |  | 7/12 | 58.3 | 0.03 | -0.26, 0.32 |  | 35/55 | 63.6 | 0.12 | -0.03, 0.27 |
| Effect of diabetes within strata of BMI |  |  | 0.49 | 0.27, 0.70 |  |  |  | 0.03 | -0.26, 0.32 |  |  |  | 0.10 | -0.04, 0.24 |

aPD, adjusted prevalence difference; BMI, body mass index; CI, confidence interval; LTBI, latent tuberculosis infection.

*P* for interaction 0.119

^a^ Adjusted for age and sex, and accounting for clustering at the family-level with an exchangeable correlation matrix.
